# Nelfinavir Inhibits Intra-Mitochondrial Calcium Influx and Protects Brain against Hypoxic-Ischemic Injury in Neonatal Mice

**DOI:** 10.1371/journal.pone.0062448

**Published:** 2013-04-22

**Authors:** Irina V. Utkina-Sosunova, Zoya V. Niatsetskaya, Sergey A. Sosunov, Veniamin I. Ratner, Dzmitry Matsiukevich, Vadim S. Ten

**Affiliations:** Department of Pediatrics, Columbia University, New York, New York, United States of America; Universidade Federal do ABC, Brazil

## Abstract

Nelfinavir (NLF), an antiretroviral agent, preserves mitochondrial membranes integrity and protects mature brain against ischemic injury in rodents. Our study demonstrates that in neonatal mice NLF significantly limits mitochondrial calcium influx, the event associated with protection of the brain against hypoxic-ischemic insult (HI). Compared to the vehicle-treated mice, cerebral mitochondria from NLF-treated mice exhibited a significantly greater tolerance to the Ca^2+^-induced membrane permeabilization, greater ADP-phosphorylating activity and reduced cytochrome C release during reperfusion. Pre-treatment with NLF or Ruthenium red (RuR) significantly improved viability of murine hippocampal HT-22 cells, reduced Ca^2+^ content and preserved membrane potential (Ψm) in mitochondria following oxygen-glucose deprivation (OGD). Following histamine-stimulated Ca^2+^ release from endoplasmic reticulum, in contrast to the vehicle-treated cells, the cells treated with NLF or RuR also demonstrated reduced Ca^2+^ content in their mitochondria, the event associated with preserved Ψm. Because RuR inhibits mitochondrial Ca^2+^ uniporter, we tested whether the NLF acts via the mechanism similar to the RuR. However, in contrast to the RuR, in the experiment with direct interaction of these agents with mitochondria isolated from naïve mice, the NLF did not alter mitochondrial Ca^2+^ influx, and did not prevent Ca^2+^ induced collapse of the Ψm. These data strongly argues against interaction of NLF and mitochondrial Ca^2+^ uniporter. Although the exact mechanism remains unclear, our study is the first to show that NLF inhibits intramitochondrial Ca^2+^ flux and protects developing brain against HI-reperfusion injury. This novel action of NLF has important clinical implication, because it targets a fundamental mechanism of post-ischemic cell death: intramitochondrial Ca^2+^ overload → mitochondrial membrane permeabilization → secondary energy failure.

## Introduction

Neonatal hypoxic-ischemic brain injury (HI) is a leading cause of permanent neurological deficit in children. The mechanisms of cerebral damage following severe HI-insult are not fully understood. Therefore, there are no mechanism-targeted strategies in the management of infants with HI encephalopathy. Mitochondria have been recognized as organelles mediating cellular injury in the HI-reperfusion brain injury [1], [2], [3]. The primary mechanism responsible for irreversible cellular damage following HI is permeabilization of mitochondrial membranes. There are two types of membranes permeabilization in post-ischemic mitochondria: Bax/Bak dependent outer membrane permeabilization and Ca^2+^ triggered, cyclophilin D-sensitive, inner membrane permeabilization, known as mitochondrial permeability transition pore (mPTP). An opening of mPTP results in a loss of the proton-motive force for ATP synthesis, and eventuates in a release of pro-apoptotic proteins [4], [5]. An opening of Bax/Bak sensitive outer mitochondrial membrane pore also causes a release of pro-apoptotic proteins. Although, studies in mature animals demonstrated that following cerebral and cardiac ischemia irreversible cell injury takes place when mitochondria open the cyclophilin-D sensitive mPTP [5], [6], [7] [8], [9], the pathogenic significance of mPTP opening in the immature HI-brain is uncertain. For example, in contrast to adult mice, neonatal cyclophilin-D knock-out mice were susceptible to HI-injury [10]. An antagonist of cyclophilin-D, cyclosporine A, did not alter the extent of HI-brain damage in neonatal rats [11]. However, using the same model Hwang et al reported that cyclosporine A, injected immediately after HI-insult protected developing brain, attenuating both necrotic and apoptotic cell death in neonatal rats [12]. Similar results were obtained in neonatal rats subjected to a mild focal cerebral ischemia-reperfusion [13]. In neonatal rats and mice subjected to a global hypoxia-ischemia-reperfusion injury, post-treatment with cyclosporine A markedly potentiated neuroprotective effect of Ca^2+^ channel antagonist, nimodipine [14].

Recently, Weaver at al. have shown a robust neuroprotective effect of antiretroviral agent, Nelfinavir (NLF) in the model of a focal ischemic injury in mature rodents [15]. In this report the therapeutic effect of NLF was attributed to a partial prevention of mPTP opening mediated by inhibition of adenine nucleotide translocator (ANT). In Jurkat T-cells NLF inhibited mitochondria-dependent apoptosis by preventing a loss of mitochondrial membrane potential and subsequent cytochrome C release [16]. It has been shown that NLF partially prevented mitochondrial outer membrane permeabilization, reducing the release of pro-apoptotic proteins from mitochondria [17]. These studies suggest that NLF is able to alter the process of outer and inner mitochondrial membranes permeabilization, both of which have been proposed as mechanisms of brain damage following perinatal HI-insult [2], [3], [10]. Our study was designed to determine whether NLF protects neonatal brain against HI-insult. Mechanistically, this study was focused on an inhibiting effect of NLF in Ca^2+^ triggered opening of mPTP during post-HI reperfusion.

## Materials and Methods

### Ethics Statement

In accordance with the National Institute of Health Guidelines for animal research, all animal procedures for these experiments were reviewed and approved by the Institutional Animal Care and Use Committee of the Columbia University (Protocol # AC-AAAA6294). Surgical procedures were performed under isoflurane anesthesia. Injections, functional testing and live handling of mice were done with all efforts to minimize distress.

### Animals and Study Groups

In all experiments we used neonatal, 9–10 days old (P9– P10) C57Bl/6J mice purchased at P5 with their dams (Jackson Laboratory, ME). The following study groups were formed: 1) Control group (naïve), mice with no interventions performed; 2) NLF-treated group; mice pre-treated with NLF and exposed to HI and 3) Vehicle-treated group; mice pre-treated with vehicle and exposed to HI.

#### The model of HI and NLF administration

HI-insult was produced according to the Rice-Vannucci model of HI-brain injury adapted to P9–10 neonatal mice [18], [19]. The pathophysiology of the brain injury in this model is ischemia-reperfusion [20]. Briefly, mice were anesthetized with isoflurane (2 vol % for induction and 0.8 vol % maintenance) and a permanent ligation of the right carotid artery was made under dissecting microscopy. After ligation pups were returned to their dams and were kept at 32°C for 90 minutes. Then mice were exposed to hypoxic insult (humidified 8% O_2_+92% N_2_) for 15 min in the hypoxic chamber placed in the temperature-controlled (37°C) neonatal isolette (Airshield Inc. NC). After hypoxic exposure pups were returned to their dams and were kept overnight in the isolette at 32°C to minimize a temperature-related variability in the extent of brain injury [19].

NLF was administered by intraperitoneal injection of 50 µl of solution (200 µg/g NLF dissolved in 4% DMSO, 5% PEG-400, 5% Tween-80 in 0.9% sodium chloride) at 20 hours prior to HI-insult. This dose was adapted from the study that demonstrated neuroprotective effect of the agent in adult mice with focal stroke [15]. 50 µl of the Nelfinavir solvent was used as vehicle.

**Quantification of brain infarct volume** was performed in 24 hours after HI as described previously [18]. Briefly, mice were sacrificed by decapitation. Brains were sectioned into 1 mm thick coronal slices and incubated in 2% TTC (2, 3, 5-triphenyl-tetrazolium chloride). Digital images were analyzed with NIH image 1.62 software by the investigator ‘blinded’ to a study groups. The area of the brain infarct was quantified and expressed as a percentage of the hemisphere ipsilateral to the carotid artery ligation side.

**Mitochondria isolation** was done as described before [21]. Briefly, mice were euthanized by decapitation. Brain samples were homogenized in the isolation buffer (225 mM Mannitol, 75 mM Sucrose, 5 mM HEPES, 1 mM EGTA (pH 7.2), 0.1 mg/ml BSA) and centrifuged at 1,100 g for 3 min at 4°C. The supernatant (750 µl) was mixed with 70 µl of 80% percoll, overlayed on 10% percoll (700 µl) and centrifuged at 18,500 g for 15 min. Obtained mitochondrial pellet was washed twice in sucrose buffer (250 mM Sucrose, 5 mM HEPES, 100 µM EGTA (pH 7.2), 0.1 mg/ml BSA) and centrifuged at 10,000 g for 5 min at 4C. The final pellet was resuspended in sucrose buffer w/o BSA and used for functional assays.

**Mitochondrial respiration** was examined using the Clark electrode (Oxytherm; Hansatech, USA). In brief, 0.05 mg of mitochondrial protein were added to 0.5 ml of the respiration buffer composed of 200 mM sucrose, 25 mM KCl, 2 mM K_2_HPO_4,_ 5 mM HEPES-KOH (pH 7.2), 5 mM MgCl_2_, 0.2 mg/ml of BSA, 30 µM Ap_5_A (*P* ^1^, *P* ^5^-di(adenosine 5′)-pentaphosphate - an inhibitor of adenylate kinase). The buffer was supplemented with substrates, 10 mM sodium glutamate, and 5 mM sodium malate. ADP (100 nmoles) was added to initiate a phosphorylating respiration (State 3). Rates of O_2_ consumption were expressed in nmol O_2_/mg mitochondrial protein/min. 70 µM 2′-4′dinitro-phenol (DNP) was used to estimate a maximal respiration rate.

**Mitochondrial calcium buffering capacity** was measured with the fluorescent, membrane impermeable dye 5N-Calcium Green as described [10], [22] with minimal modifications established for fluorimeter Hitachi 7000 (Ex 488 nm and Em 531 nm). Briefly, mitochondria (0.05 mg/ml) were incubated in 10 mM Tris-MOPS buffer (pH 7.4), containing 120 mM KCl, 1 mM K2HPO4, 10 µM EGTA, 5 mM sodium succinate, 2.5 mM sodium glutamate, and 1 µM 5N-Calcium Green. Mitochondria were tested with 10 nmoles of CaCl_2_ pulses repeatedly added every 50 seconds until mitochondrial membranes permeabilization was detected as spontaneous release of calcium. The amount of calcium required for mPTP opening was measured in nmoles/mg of mitochondrial protein.

For assessment of direct influence of the NLF on mitochondrial calcium uptake, mitochondria were isolated from naïve mice. Initially, mitochondria (0.05 mg/ml) were tested for Ca^2+^ uptake ability. Then, the same mitochondria were incubated with 4.4 µM of NLF for 5 min in the cuvette, followed by Ca^2+^ pulses (10 nmoles). Ruthenium Red (RuR, 1 µM), an inhibitor of mitochondrial calcium uniporter, was used in the same experimental paradigm.

**Mitochondrial membrane potential (Ψm)** was measured using fluorescent dye, Safranin (1 µM) which quenches fluorescence interacting with intact Ψm. Briefly, once the baseline of the Safranin fluorescence in the buffer (10 mM Tris-MOPS buffer (pH 7.4), 120 mM KCl, 1 mM K2HPO4, 5 mM sodium succinate, 2.5 mM sodium glutamate, 0.2 mg/ml BSA, 1 mM ATP) was reached, mitochondria (0.05 mg/ml) was added and an immediate quenching in the Safranin fluorescence was recorded. Once the minimal Safranin fluorescence was reached, at 100 seconds following supplementation with mitochondria, 50 nmoles of Ca pulses were given every 50 seconds until the Safranin fluorescence reached the baseline again. At this point, the absence of any changes in Safranin fluorescence following supplementation with carbonyl cyanide 4-(trifluoromethoxy)phenylhydrazone (FCCP, 60 nM) confirmed that the Ψm is fully collapsed. Mitochondrial samples were pre-incubated for five minutes either with NLF (4.4 µM) or RuR (1 µM) or vehicle (0.001% DMSO in the respiratory buffer).

***Ex-vivo*** **quantification of mitochondrial calcium load** was determined with fluorescent dye, 5N-Calcium Green. Briefly, mitochondria were isolated without EGTA. Mitochondria (0.1 mg/ml) were incubated in 10 mM Tris-MOPS buffer (pH 7.4), containing 120 mM KCl, 1 mM K2HPO4, 10 µM EGTA, 5 mM sodium succinate, 2.5 mM sodium glutamate, 1 µM 5N-Calcium Green. Following 200 sec of the stabilization of fluorescence, 0.1% digitonin was added to disrupt mitochondrial membranes and to release Ca^2+^. An increase in calcium fluorescence was recorded until it reached a plateau. The amount of the released mitochondrial calcium was quantified as an increase in the Ca^2+^ fluorescence normalized to the digitonin-induced non-specific fluorescence and expressed in nmoles per mg of mitochondrial protein.

#### Assessment of mitochondrial calcium, membrane potential and viability in HT-22 cells

Mouse hippocampal neural/glial immortalized HT-22 cell line (Lonza, Walkersville, MD) was grown in Dulbecco’s modified Eagle’s medium (DMEM), supplemented with 25 mM glucose, 2 mM L-glutamine, 2 mM pyruvate, penicillin (10 units/ml), streptomycin (10 mg/ml), and 10% (vol/vol) heat inactivated fetal bovine serum (FBS) in a humidified cell incubator (Binder, Germany) at 37°C under a 5% CO_2_ atmosphere.

Oxygen-Glucose Deprivation (OGD) was performed in DMEM (glucose and pyruvate-free) with 1% (vol/vol) FBS. Hypoxic (O_2_<0.1%) level was maintained for 12 hours by continuous flow of N_2_ 95% and 5% CO_2_ under automatic O_2_ and CO_2_ control. Normoxic incubation was done in the DMEM (pyruvate-free) containing 25 mM glucose at 37°C under a 5% CO_2_/21% O_2_.

Histamine-induced Ca^2+^ release from endoplasmic reticulum into cytosol was performed as described [23]. Nelfinavir (4.4 µM) or ruthenium red (10 µM) or Cyclosporin A, CsA (1 µM) or vehicle (0.001% DMSO in DMEM for NLF, DMEM for RuR, 0.00001% ethanol in DMEM for CsA were added at 20 hours prior to the OGD or histamine (10 mM) challenges. The nelfinavir dose was selected to avoid cellular apoptosis observed at higher doses [16]. For live imaging cells were plated at a density of 10×10^3^ cells/cm^2^ into 30 mm culture dishes with 10 mm glass bottom inserts (MatTek Inc., Ashland, MA). Calcium was detected with fluorescent dye, Rhod-2 (0.5 µM) for mitochondrial Ca^2+^ content or Fluo 4 (1 µM) for total cellular Ca^2+^ content. Mitochondrial membrane potential (Ψm) was assessed with fluorescent probes, Rhodamine 123 (R123, 1 µM) or tetra-methylrhodamine, ethyl ester perchlorate, (TMRE, 25 nM). Cells were examined under NIKON A1R MP confocal microscope equipped with temperature, gas and humidity control station for live-cell studies. Semi-quantitative analysis of the total cellular or mitochondrial Ca^2+^ content and Ψm was performed based on optical density (OD) of the merged images (stack of 5 optical slices obtained at a step of 0.25 µm, 1024×1024 pixel resolution, observed area 295×295 µm) with Image-J software (public domain). All data were expressed in arbitrary fluorescent units (FU). Changes in the Ca^2+^ and Ψm specific fluorescence in response to histamine or OGD challenge were expressed in % in the relation to controls (mean = 100%). Assessment of cellular viability was performed at six hours following OGD. Cells were stained with propidium iodide (PI, 10 ug/ml) and Hoechst (10 ug/ml). Amount of PI positive cells and a number of nuclei were calculated in the images (stack of 5 optical slices obtained at a step of 0.5 µm, 1024×1024 pixel resolution, observed area 644×644 µm). The cellular mortality was estimated as a ratio of PI positive cells to a total number of cells detected with Hoechst. Each experiment for live imaging was repeated at list four times and at list of 15 images have been taken from each dish for quantitative analysis.

#### Western blotting analysis

Cytosolic fractions were obtained during mitochondrial isolation. The degradation of cytosolic proteins was preserved using 0.5% protease inhibitor cocktail. Samples were run on 12% polyacrilamide NuPAGE Bis-Tris gels and transferred to 0.22 µn nitrocellulose membranes. After blocking in 5% nonfat milk membranes were probed with mouse anti-cytochrome C (BD Pharmigen, USA, 1∶2000), mouse anti-β-actin-HRP-conjugated (Sigma, USA, 1∶100,000), or mouse anti-COX IV (Abcam, USA, clone 20E8, 1∶5000) primary antibodies followed by incubation with secondary peroxidase-conjugated donkey anti-mouse antibodies (Jackson Immunoresearch, USA). The visualization of bands was performed using ECL-plus western blotting detection system (GE, Healthcare). Images were quantified and analyzed using Image-J software. Bands intensity was normalized to β-actin in the cytosolic fractions and expressed in Optical Density (OD) Arbitrary Units.

**Chemicals and reagents** were obtained from Sigma, St. Louis, CA, USA, except of D-sucrose; 0.9% sodium chloride; MOPS; K2HPO4 (Fisher Scientific**,** Agawam, MA, USA); 5N-calcium green; Rhod-2; Fluo-4; TMRE; Rhodamine 123; Mitotracker green; Hoechst; Dimethyl sulfoxide (DMSO); 12% polyacrylamide NuPAGE Bis-Tris gels; DMEM; Fetal bovine serum (FBS) heat inactivated; penicillin; streptomycin; (Invitrogen, Carsbad, CA, USA). Nelfinavir was a generous gift from Dr. Dennis PA, Center for Cancer Research, National Cancer Institute, Bethesda, Maryland, USA.

### Statistical Analysis

Student’s T-test, one-or two ways ANOVA or ANOVA for repeated measures with Fisher’s post-hoc analysis were used when appropriate. All data were present as mean ± SE. A difference was considered significant with p ≤ 0.05.

## Results

### Nelfinavir Decreases Intramitochondrial Ca^2+^ Overloading, Preserves Mitochondrial Phosphorylating Activity, and Ca^2+^ Buffering Capacity Following HI

**Figure 1:**
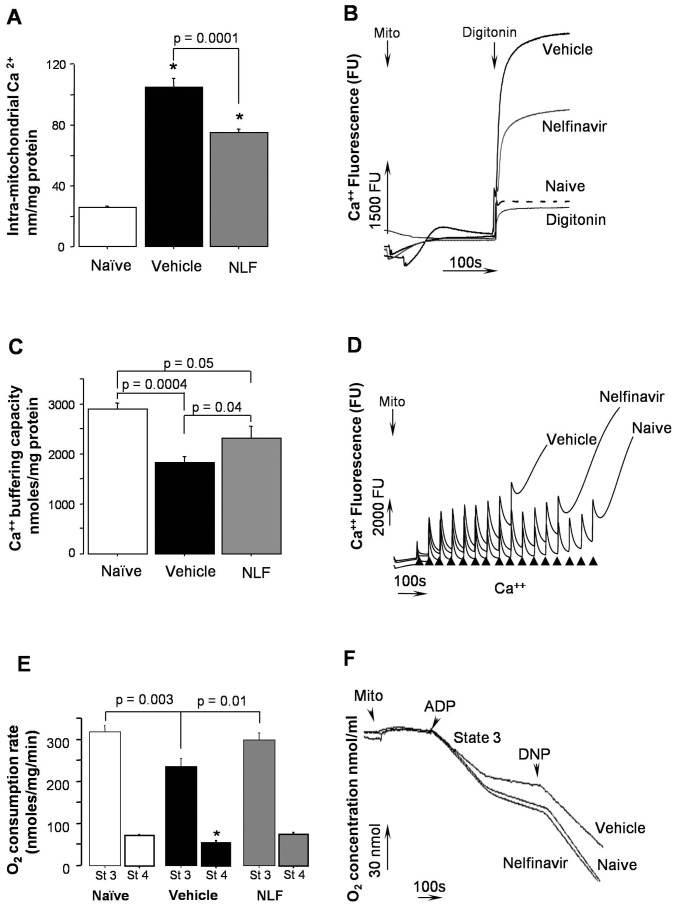
Nelfinavir improves mitochondrial function. **A, B** – Intramitochondrial Ca^2+^ content (**A**) and representative tracings of Ca^2+^ release from mitochondria (**B**) in naïve (n = 4) and at the end of HI-insult in vehicle (n = 7) or NLF-treated (n = 7) mice. One-way ANOVA. *- p<0.0001 compared to naives. Mito (downward arrow) indicates addition of mitochondria (0.1 mg/ml). Digitonin (downward arrow) shows addition of Digitonin (10 mg/mg of the mitochondrial protein). Digitonin-induced nonspecific fluorescence curve (without mitochondria addition) is indicated as Digitonin. **C, D** – Mitochondrial Ca^2+^ buffering capacity at five hours of reperfusion (**C**), with representative tracings (**D**) in vehicle (n = 11) and NLF-treated (n = 14) HI-mice compared to naïve littermates (n = 7). Mitochondrial Ca^2+^ buffering capacity was defined by the amount of Ca^2+^ needed to open mPTP (spontaneous increase in Ca^2+^ fluorescence). **E, F** - Mitochondrial ADP-phosphorylating (state 3) and resting (state 4) respiration rates (E), with representative tracing (F) examined in naïve (n = 11) and at five hours of reperfusion in vehicle (n = 16) or NLF-treated mice (n = 15). * p<0.02 compared to Naïve and NLF treated mice.

At 0 minute of reperfusion, immediately at the end of HI, brain mitochondria isolated from mice pre-treated with NLF demonstrated significantly decreased Ca^2+^ content compared to the vehicle-treated littermates (Fig. 1A, B). At five hours of reperfusion, the time-point when secondary energy failure occurs in this model [1], [2], [19], cerebral mitochondria from the NLF-treated group exhibited a significantly greater mitochondrial tolerance against Ca^2+^ induced opening of mPTP compared to that in the vehicle treated mice (Fig. 1C, D). At the same time of reperfusion, mitochondria isolated from the NLF-treated HI-mice exhibited a better-preserved ADP-phosphorylating activity, evidenced by a significantly greater state 3 respiration rate, compared to the vehicle-treated mice. State 4 respiration rate also was significantly greater preserved in the NLF-treated mice compared to the vehicle-treated mice (Fig. 1E and F). As expected, no difference was detected in the respiratory control ratio between these groups of mice (data not shown).

### Nelfinavir Limits Cytochrome c Release and Attenuates the Extent of Brain Injury

**Figure 2:**
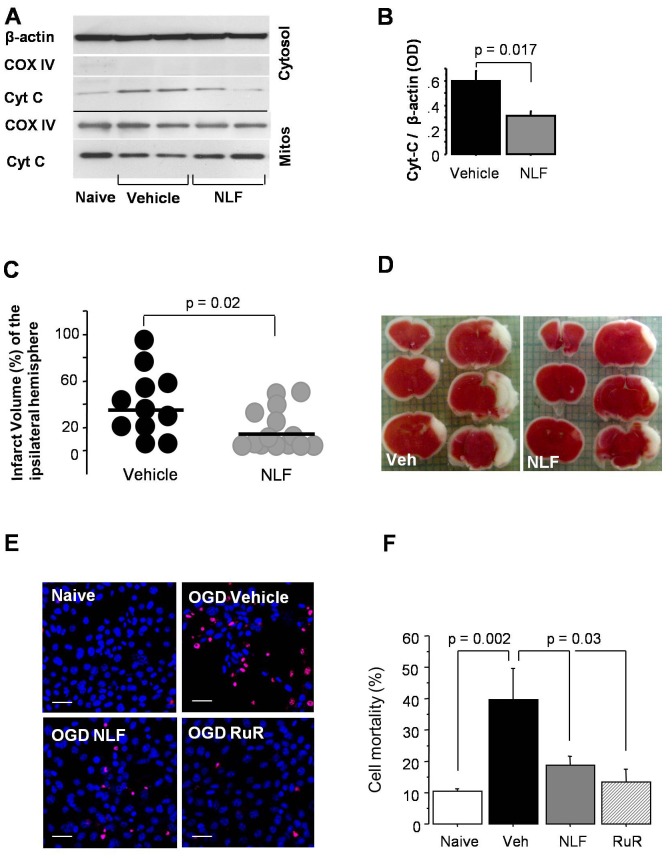
Nelfinavir limits post-ischemic mitochondrial cytochrome C release, brain infarct volume and cellular mortality. **A, B** - Western blot analysis for the presence of Cytochrome C (Cyt C) in cytosolic (Cytosol) and mitochondrial (Mitos) fractions obtained from the ipsilateral hemisphere at five hrs after HI insult. β-actin was used as a loading control and cytochrome C oxidase (COX IV) was used as a purity control of cytosolic fraction and loading control for mitochondrial fraction. Quantitative data presented in (B) are expressed in arbitrary OD units normalized to β-actin. Vehicle –treated mice, n = 3, NLF-treated mice, n = 4. **C, D** - Cerebral infarct volume and representative TTC-stained brain slices obtained at 24 hrs of reperfusion after HI in the vehicle and NLF-treated mice. **E, F** – Cell mortality at 6 hours of reperfusion following 12 hours of OGD in HT-22 cells treated with vehicle (n = 7), NLF (n = 7) or RuR (n = 7) compared to naives (n = 4). **E** - Representative images of HT-22 cells in different experimental conditions stained with Propidium Iodide (red) and Hoechst (blue). Note that amount of red cells predominates in OGD-vehicle group. Confocal microscopy. Scale bar = 50 µm. **F** - Quantitative evaluation of cell mortality. One-way Anova, only significant difference is shown.

At five hours of reperfusion, pre-treatment with NLF resulted in significantly reduced release of cytochrome c from the post-ischemic brain mitochondria (Fig. 2A, B). To this point our data demonstrate that compared to the vehicle, the pre-treatment with NLF (a) significantly decreased Ca^2+^ content in cerebral mitochondria at the end of HI-insult, (b) improved mitochondrial tolerance against Ca^2+^ induced permeabilization of mitochondrial membranes in reperfusion, associated with (c) partially preserved ADP-phosphorylating activity, and (d) a better-preserved mitochondrial membrane integrity, evidenced by the decreased release of the cytochrome c from mitochondria. All these effects of NLF pre-treatment were coupled with a significant reduction of the infarct volumes in mice pre-treated with NLF compared to the vehicle-treated littermates (Fig. 2C, D).

### Nelfinavir Prevents Mitochondrial Ca^2+^ Influx and Mimics the Effect of Ruthenium Red

**Figure 3:**
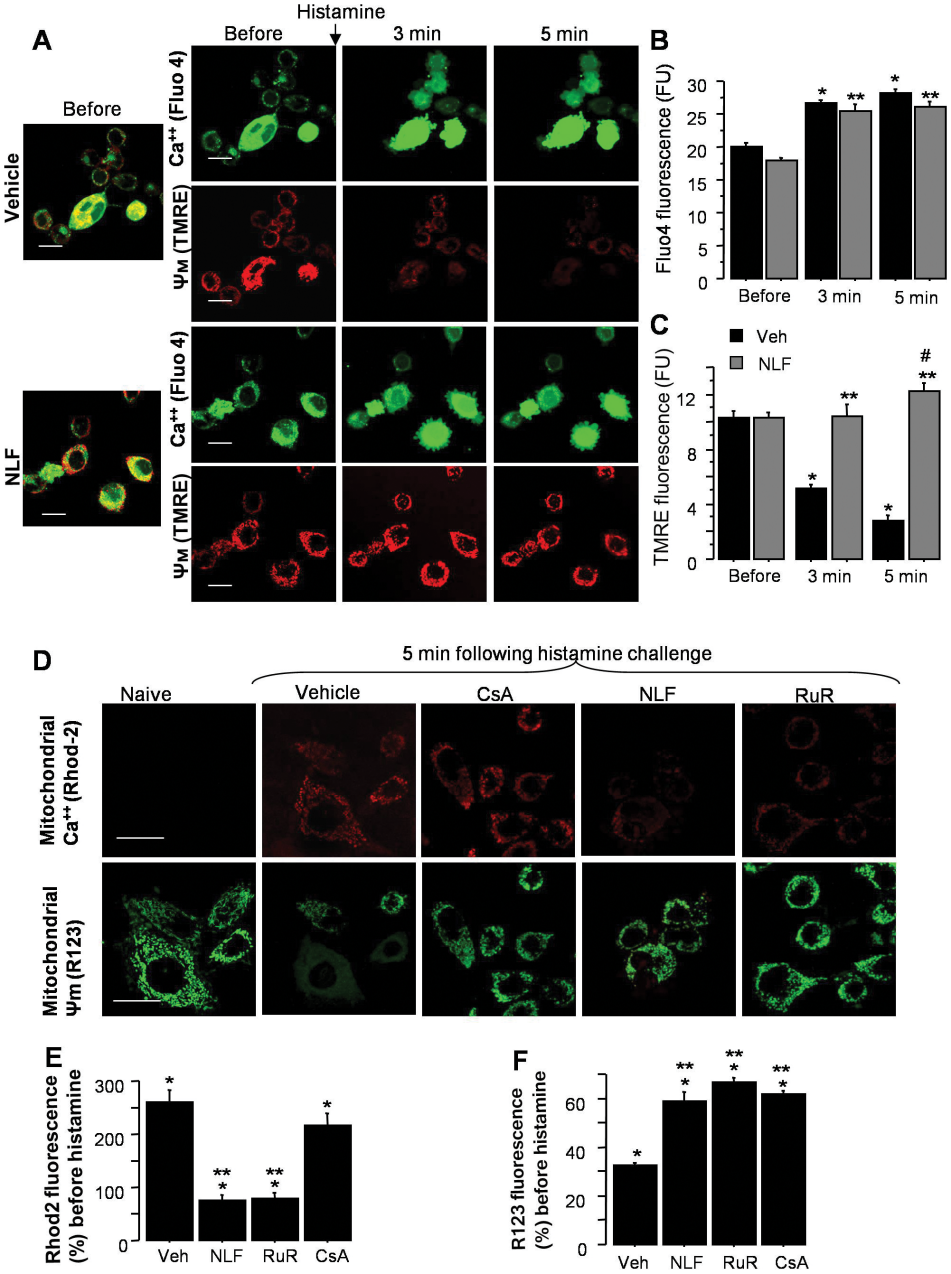
Nelfinavir mimics the effect of RuR on mitochondrial response to histamine. **A–C**, - Confocal microscopy and semi-quantitative analysis of cytosolic cellular Ca^2+^ fluorescence (Fluo4) with simultaneous Ψm fluorescence (TMRE) in cells treated with vehicle (n = 6) or NLF (n = 6), before and after histamine (10 mM) challenge. * p<0.0001 compared to values before histamine challenge in the vehicle, and ** p<0.0001 in the NLF treated cells. **D–F,** - Confocal microscopy and semi-quantitative analysis for mitochondria-specific Ca^2+^ (Rhod 2) fluorescence alone with Ψm (R123) fluorescence in cells pre-incubated (20 hrs) with NLF (4.4 µM), RuR (10 µM) or CsA (1 µM). * p<0.0001 compared to naives, ** p<0.0001 compared to the vehicle-treated cells. n = 6 in each group. Scale bars = 10 µm and 20 µm.

**Figure 4:**
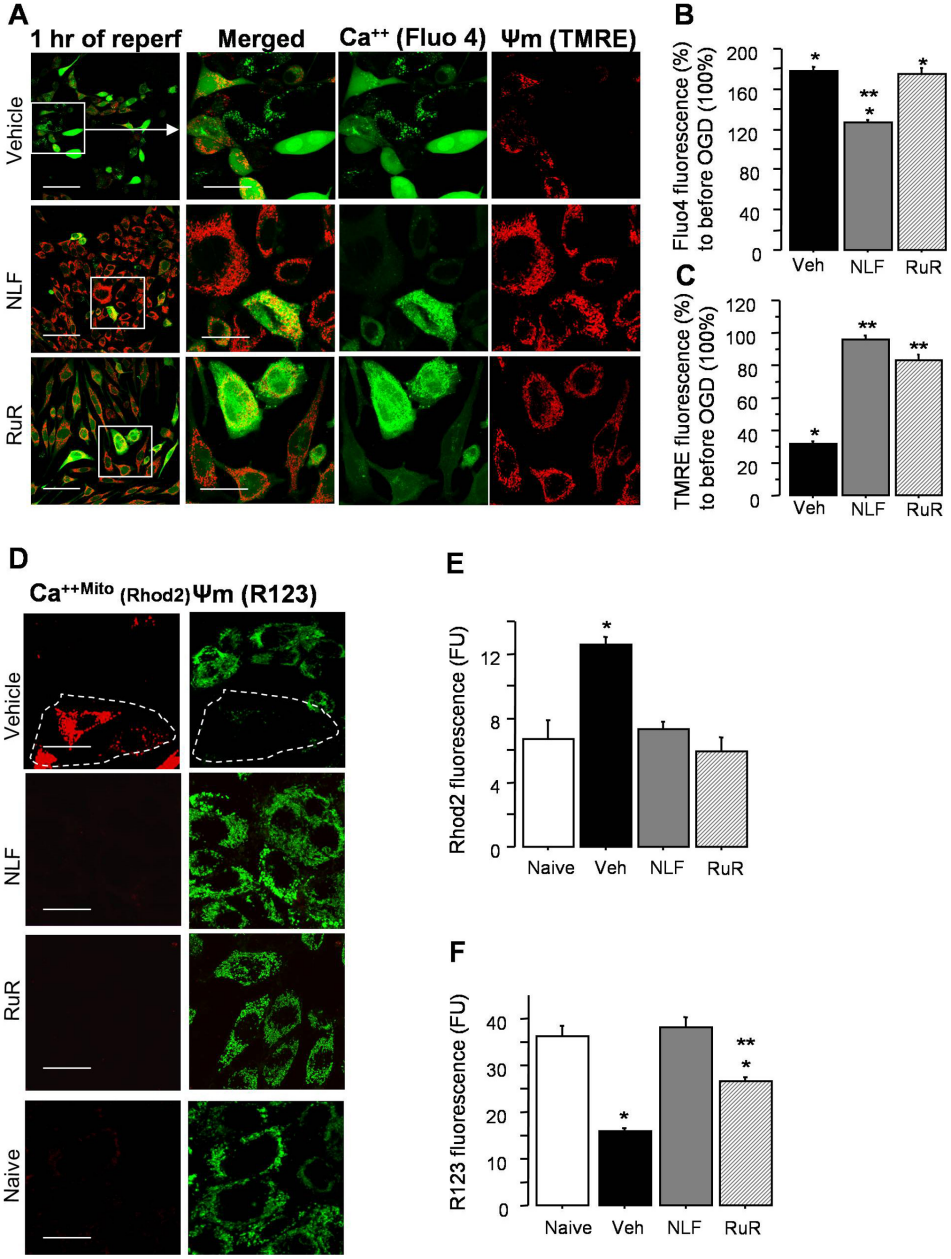
Nelfinavir mimics the effect of RuR on mitochondrial response to OGD. **A–C,** - Confocal microscopy and semi-quantitative analysis of cytosolic cellular Ca^2+^ fluorescence (Fluo4) with simultaneous Ψm fluorescence (TMRE) in cells treated with vehicle (n = 7) or NLF (n = 7), or RuR (n = 7) at 1 hrs following OGD (12 hrs). * p<0.0001 compared to values compared to the naïve cells (100%), and ** p<0.0001 compared to the vehicles. **D–F** -Confocal microscopy and semi-quantitative analysis for mitochondria-specific Ca^2+^ (Rhod 2) fluorescence alone with Ψm (R123) fluorescence in naïve cells and cells pre-incubated (20 hrs) with vehicle or NLF (4.4 µM), RuR (10 µM). * p<0.0001 compared to naives, ** p<0.0001 compared to the NLF cells. n = 7 in each group. The area outlined with dashed line demonstrates cells with Ca^2+^overloaded mitochondria which lost their Ψm. Scale bar = 40 (merged images) and 20 µm.

**Figure 5:**
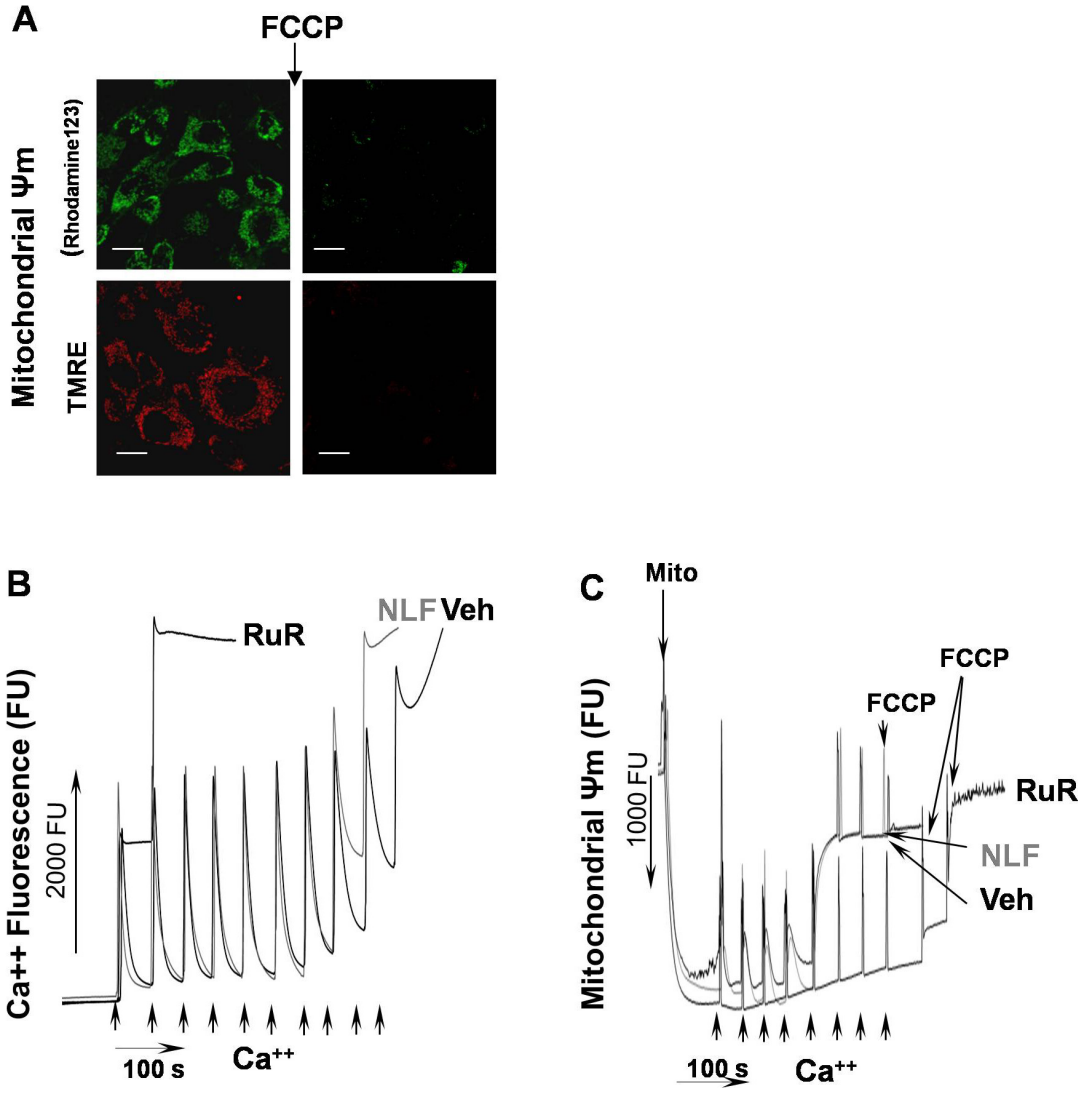
Nelfinavir does not inhibit mitochondrial Ca^2+^ uniporter. **A** – The experiment controlling mitochondrial specificity for TMRE and Rhodamine 123 (R123) fluoroprobes. Note a drastic decrease in Ψm fluorescence following FCCP (0.5 µM) supplementation. Scale bar = 10 µm. **B** – One of three highly reproducible tracings of mitochondrial Ca^2+^ buffering capacity in organelles pre-treated with RuR (1 µM), NLF (4.4 µM) or vehicle. Note, that only RuR completely inhibited Ca^2+^ up-take by mitochondria, while NLF, virtually, had no effect. **C** – One of the four highly reproducible tracings of changes in the safranin (Ψm) fluorescence in response to mitochondrial supplementation (indicated) and addition of 10 nmoles of Ca^2+^ pulses to mitochondria pre-incubated with RuR, NLF or vehicle. Note, only the RuR prevented the collapse of Ψm in response to Ca^2+^ challenge. Brain mitochondria were isolated from naïve p10 mice, substrate: succinate-glutamate (see also methods).

Based on these data we hypothesized that NLF prevents intra-mitochondrial Ca^2+^ influx via inhibition of mitochondrial Ca^2+^ uniporter channel. Firstly, we examined whether an inhibition of mitochondrial Ca^2+^ up-take protects cells against an ischemic insult. Fig. 2E and F demonstrate that compared to the vehicle-treated HT-22 cells in presence of NLF or ruthenium red (RuR), known inhibitor of mitochondrial Ca^2+^ uniporter, exhibited a significantly greater viability following OGD challenge. When HT-22 cells were exposed to histamine, both groups of cells demonstrated the same extent of a significant increase in their cytosolic Ca^2+^ content compared to that prior to histamine challenge (Fig. 3A and B). However, those cells that were pre-treated with NLF significantly greater preserved Ψm compared to the vehicle-treated counterparts (Fig. 3 A and C). Simultaneous tracing of mitochondrial Ca^2+^ content and Ψm in the same cells, revealed that compared to the vehicle-treated cells, NLF or RuR treated cells exhibited reduced intramitochondrial Ca^2+^ fluorescence, the event associated with preserved Ψm (Fig. 3D–F). The Cyclophylin D dependent nature of this Ca^2+^-triggered Ψm collapse was controlled by the use of Cyclosporine A. Cyclosporine A significantly preserved Ψm, in spite of a similar mitochondrial Ca^2+^ load compared to vehicle-treated cells (Fig. 3D–E). Importantly, similar alteration of mitochondrial response to an ischemia-reperfusion mimicking paradigm was observed in the presence of RuR or NLF. Following OGD challenge, all groups of HT-22 cells exhibited significant increase in their cytosolic Ca^2+^ fluorescence compared to controls (Fig. 4A, B). However, in the presence of RuR or NLF cells maintained their Ψm, while in the vehicle-treated cells the Ψm was collapsing in line with their cytosolic Ca^2+^ load (Fig. 4A–C). The preservation of the Ψm in NLF or RuR-treated OGD cells was associated with significantly reduced intramitochondrial Ca^2+^ content (Fig. 4D–F). Interestingly, the extent of Ψm preservation was significantly greater in the NLF-treated cells compared to the RuR-treated counterparts, although mitochondrial Ca^2+^ content in these cells was comparable (Fig. 4D–F). The total Ca^2+^content, however, was significantly decreased in NLF vs RuR exposed cells (Fig. 4B). The specificity of the Ψm fluorescence was controlled by the use of mitochondrial uncoupler, FCCP which, as expected, collapsed the Ψm in naïve cells (Fig. 5A).

### Nelfinavir does not Affect Mitochondrial Ca^2+^ Uniporter

Although, our in vitro data demonstrate a stunning similarity in the phenotype of mitochondrial response to OGD or histamine challenge between cells treated with RuR or NLF, the experiment testing the inhibiting action NLF on Ca^2+^ uniporter in isolated mitochondria refuted our hypothesis. Fig. 5B and C clearly demonstrate that, in robust contrast to the RuR effects, the NLF did not block mitochondrial Ca^2+^ influx and failed to preserve Ψm collapsed by the Ca^2+^.

## Discussion

There are two important results: (1) pre-treatment with NLF significantly reduced the HI-brain injury in neonatal mice and (2) this neuroprotection was associated with attenuation of Ca^2+^ influx into cerebral mitochondria during HI-insult and reperfusion.

Mitochondrial calcium overload during and, especially, following cerebral or cardiac ischemia has been considered as one of the key pathogenic events in the post-ischemic cellular death pathway [24] [7], [8]. Mitochondrial Ca^2+^ overload results in a loss of the mitochondrial membrane integrity secondary to an opening of mitochondrial permeability transition pore. An opening of mPTP dissipates the H^+^ gradient across the inner membrane, causing a loss of the proton motive force for ATP synthesis. This mechanism has been suggested to explain secondary energy failure in reperfusion [25]. mPTP also causes mitochondrial swelling with subsequent rupture of mitochondrial outer membranes and release of pro-apoptotic proteins (reviewed in [26]. Although, mPTP was discovered more than 40 years ago, the exact structure and mechanisms of mPTP formation in the post-ischemic mitochondria remain unclear. What was firmly determined, however, that Ca^2+^ influx into mitochondria is the main triggering factor in the opening of mPTP [27], [28], [29]. Our finding that brain mitochondria pre-exposed to NLF *in vivo* contained significantly less Ca^2+^ at the end of the HI-insult is very important, because the organelles preloaded with Ca^2+^ during HI-insult quickly exhaust their Ca^2+^ buffering capacity during reperfusion when the major Ca^2+^ influx into mitochondria takes place [6], [25]. Indeed, at five hours of reperfusion, the time-point when secondary energy failure occurs in this model [1], mitochondria isolated from the mice pre-treated with NLF exhibited a significantly greater Ca^2+^ buffering capacity, the parameter which defines a threshold of mitochondrial resistance against permeabilization of their membranes. A better-preserved mitochondrial membrane integrity in mice treated with NLF could be supported with data showing a markedly reduced cytochrome C release into cytosol in the mice treated with NLF, compared to that in the vehicle-controls. This better-preserved cytochrome C content in the mitochondria may account for a significantly better-preserved ADP-phosphorylating activity compared to the vehicle-treated counterparts. It has been shown that an opening of mPTP resulted in substantial loss of the cytochrome C, associated with significant inhibition of ADP-phosphorylating activity which was prevented by the cyclosporine A [30], [31]. Given, that NLF limits mPTP formation, the severity of post-HI mitochondrial dysfunction (secondary energy failure) could be alleviated by the pre-treatment with NLF. One of the mechanisms for this NLF-driven attenuation of the severity of secondary bioenergetics crisis is the prevention of mitochondrial Ca^2+^ overload, the event upstream of the key cell-death mechanism – an opening of mPTP.

One of the most important channels responsible for the Ca^2+^ influx into mitochondria is Ca^2+^ uniporter [32]. We hypothesized that NLF alters the function of the Ca^2+^ uniporter. We have employed an “analogy approach” to obtain evidence to support this hypothesis. Our experiments with the use of HT-22 cells demonstrated a stunning similarity in mitochondrial response to the cytosolic Ca^2+^ overload in the presence of NLF or RuR. Of note, cellular Ca^2+^ overload was induced by two different paradigms; the OGD or histamine challenge. In both experimental settings the NLF or RuR significantly limited Ca^2+^ load to mitochondria and prevented a collapse of Ψm secondary to the cyclosporine A-sensitive mPTP opening. However, in the experiments with isolated mitochondria, NLF exerted absolutely no effect on mitochondrial Ca^2+^ upload and, most importantly, did not prevent a loss of the Ψm in response to Ca^2+^. In contrast, as expected, RuR fully preserved Ψm during Ca^2+^ challenge and completely blocked mitochondrial Ca^2+^ up-take. Thus, our data demonstrate that in vivo and in vitro the pre-treatment with NLF significantly limits intramitochondrial Ca^2+^ flux, but the mechanism of this novel pharmacological action is unrelated to the inhibition of the mitochondrial Ca^2+^ uniporter. It has been reported that NLF limits intracellular Ca^2+^ flux in beta-cells [33]. This effect of NLF may partially account for a significantly decreased total Ca^2+^ content following OGD in the cells pre-treated with NLF compared to that pre-treated with RuR (Fig. 4B). However, in the experiment with histamine-driven Ca^2+^ release from endoplasmic reticulum, the total cytosolic Ca^2+^ content was similar in the NLF or RuR exposed cells. Yet, the intra-mitochondrial Ca^2+^ load was significantly decreased in the presence of both agents. This result strongly suggests that NLF attenuates intra-mitochondrial Ca^2+^ flux when cells are overloaded with Ca^2+^.

Although, the exact mechanism for the NLF-driven inhibition of mitochondrial Ca^2+^ up-take remains to be determined, to the best of our knowledge, this is the first report demonstrating that NLF protects the developing brain mitochondria against HI-induced membrane permeabilization by limiting mitochondrial Ca^2+^ influx. In the adult rodent model of focal brain ischemia, Weaver at al have reported that the pre-treatment with NLF inhibited participation of adenine nucleotide translocator (ANT) subunit in the formation of the mitochondrial permeability transition pore complex [15]. Although there is a line of indirect evidence consistent with an important role of the ANT in mPTP opening [34], genetic ablation of the ANT has revealed that this protein is not essential for mPTP formation [35]. It is possible, however, that NLF limits a functional assistance of ANT in the opening of the Ca^2+^-induced mPTP, since ANT1 and ANT2 knock-out mouse liver mitochondria were less sensitive to Ca^2+^ compared to wild-type mitochondria. Regardless whether NLF interacts with ANT or not, the inhibiting effect of NLF on intra-mitochondrial Ca^2+^ flux cannot be overestimated, because Ca^2+^ overload is the primary trigger for an opening of mPTP in the ischemia and reperfusion.

Thus, our study shows that inhibition of Ca^2+^ influx into mitochondria during and following ischemia represents a potential neuroprotective strategy, targeting the key event: mitochondrial membrane permeabilization in the cell death pathway.
